# Vision zero: a toolkit for road safety in the modern era

**DOI:** 10.1186/s40621-016-0098-z

**Published:** 2017-01-09

**Authors:** Ellen Kim, Peter Muennig, Zohn Rosen

**Affiliations:** Global Research Analytics for Population Health, Mailman School of Public Health, Columbia University, 722 West 168th Street, New York, NY 10032 USA

**Keywords:** Vision zero, Road safety, Traffic, Accidents, Crashes, Injury

## Abstract

Vision Zero (VZ) is a public program that aims to have zero fatalities or serious injuries from road traffic crashes. This article examines various major components of VZ: how VZ redefines road safety, how VZ principles and philosophies can be applied to modern car and road designs, and how VZ can be applied to traffic. Applications of these principles to real-world traffic infrastructure are explored in order to show policymakers the toolkits available to increase road safety while taking into consideration local contexts.

## Review

### Introduction

More than 1.2 million people worldwide are killed each year from road traffic crashes, and an additional 50 million people are injured (World Health Organization 2015). These crashes are often perceived as isolated incidents caused by driving errors, rather than being viewed collectively as a public health problem. However, viewing crashes as errors has led to an emphasis on enacting road safety measures that solely focus on road users (Tingvall and Haworth 1999). Certainly, road users are an important part of the road transport system, but infrastructure (including enforcement infrastructure) is also important. A “systems perspective” that takes into account the interaction of design elements and all actors in the road transport system is necessary to achieve the highest level of road safety (Elvebakk 2007). One policy that aims to do exactly that is Vision Zero (VZ), which was created in Sweden and adopted by the Swedish parliament in 1997 (Johansson 2009). While there are outstanding papers that describe the basic ideas behind VZ, a lot has been learned since and most are written for an urban planning audience. The purpose of this paper is to critically examine how VZ redefines road safety as a public health issue, to update the literature on VZ, and to explore how local context could guide its implementation.

VZ’s long-term goal, as the name suggests, is to have zero fatalities or serious injuries from road traffic “accidents” (Tingvall and Haworth 1999). While that might not be a realistic goal, its proponents believe that there is no such thing as a traffic “accident” and that every crash is avoidable. This drastically changes the way road users and policymakers view road safety.

VZ redefines road safety by taking a public health approach to collisions, i.e., that they are a preventable health threat. As such, VZ explicitly states that responsibility for road traffic collisions is shared between road users and system designers (Tingvall and Haworth 1999; Belin et al. 2012), such as transportation safety experts, educators, public health professionals, and car designers and manufacturers (McAndrews 2013). As such, system design is a collective interdisciplinary effort.

In addition to providing a new framework for thinking about road safety, VZ also provides a toolkit of design (e.g., implementation of median barriers, modern roundabouts, speed humps, pedestrian islands, curb extensions, etc.) and enforcement methods (e.g., technological improvements, installation of speed cameras, red light cameras, etc.) that make car travel safer (City of New York 2014; Walljasper 2015; http://www.visionzeroinitiative.com/; Swedish Transport Administration 2012). The design elements complement the enforcement elements, and together form a network of safety measures intended to slow traffic, provide safe havens for pedestrians, and to reduce road hazards.

This paper begins by examining the general philosophy of VZ and how VZ adopts the public health perspective on road safety. The second part of this paper explores the design principles of VZ. The last section of this paper concludes with examples of how VZ principles can be applied to real-world traffic infrastructure in order to make roads safer, considering local contexts.

### Vision Zero’s philosophy

VZ was first adopted in Sweden in 1997, when the Swedish Parliament passed a bill on traffic safety (the Road Traffic Safety Bill). Surprisingly, VZ was not implemented in response to a relatively high death rate from road traffic collisions; Sweden already had one of the lowest rates of road traffic casualties in the world. Rather, VZ was implemented because advocates argued that any deaths were too high of a price to pay for mobility (Elvebakk 2007).

This view was meant to contrast with what was perceived to be the common belief that road traffic crashes and fatalities are a “necessary evil to be accepted in the interests of personal mobility” (Tingvall and Haworth 1999). VZ emphasizes that safety and mobility cannot be weighed against each other. Instead, mobility has to become a function of safety so that greater mobility is afforded only when a road system is safe (Tingvall and Haworth 1999). This approach follows the standards set in the air, railway, and nuclear power industries, which consider any injuries or deaths as preventable tragedies (Elvebakk 2007; McAndrews 2013). VZ asserts that the road traffic industry should not be the exception to this rule (Elvebakk 2007).

Traditionally, the road user has been viewed as the central entity responsible for road traffic safety (Tingvall and Haworth 1999). Conventional crash analyses show that about 90–95% of all crashes are caused by road users (Johansson 2009), neglecting other underlying conditions that contribute to these collisions. As a result of this conventional view, most countries today pass rules and regulations that govern how road users should behave. Under this view, legal actions are typically brought against one of two road users involved in a collision (Tingvall and Haworth 1999). In contrast, VZ attempts to shift the responsibility so that all actors within the system are held accountable, causing them to become more centrally involved in road safety.

These actors include not just the users, but also automobile manufacturers, government transportation authorities, and other entities (McAndrews 2013). Their shared responsibility for road safety has significant implications for the future of road travel. For example, autonomous vehicles are becoming more popular amongst car manufacturers. Under the traditional system, risk-averse manufacturers may be inclined not to implement such technical features because they are costly. Under VZ, however, manufacturers may be held accountable for failing to implement such life-saving innovations. Specifically, VZ states that:

1. Since system designers are responsible for the design, operation, and use of the road transport system, they are also responsible for the safety of the entire road system.
2. Road users are responsible for adhering to the rules set forth by system designers when using the road transport system.
3. If road users fail to follow said rules due to lack of knowledge, approval or ability, or if injuries do occur, then system designers are responsible for taking further action to prevent people from being killed or seriously injured (Tingvall and Haworth 1999).

While under VZ principles, drivers and designers share liability. However, drivers are still responsible for driving safely since they assume significant risk, and drivers need to comply with any traffic regulations to ensure the safety of the road transport system.

Since VZ was first adopted by Sweden, it has grown into a global vision. Countries such as Norway, the United Kingdom, and Canada have all incorporated VZ’s philosophies into their road safety plans. Multiple cities in the United States have also adopted VZ, including New York City, Austin, Boston, San Francisco, Seattle, Portland, and San Diego. While all of these communities strive to fulfill VZ’s goals, they have implemented the program in a variety of ways to meet their own needs and means. Each country or city that adopts VZ has a unique culture and infrastructure, thus system designers need to take into consideration the existing surrounding environment before implementing the program.

### A public health perspective on road safety

Road safety is increasingly being considered a public health problem. The World Health Organization has labeled road traffic collisions as a major global health hazard as it is one of the leading and fastest growing causes of disability and death (World Health Organization 2015). It is increasingly becoming common to refer to injuries from these traffic crashes as an “epidemic” (Elvebakk 2007). Indeed, traffic crashes are now the number one cause of death for people aged 15–29 years (World Health Organization 2015). By considering crashes an epidemic, VZ aims to give the same type of attention that communicable diseases, such as malaria and polio, receive worldwide.

By making the case that most road traffic “accidents” are not actually accidental, but are predictable and preventable (Elvebakk 2007), road safety measures become a shared responsibility between transportation and public health officials. This way, a traffic crash that does not result in loss of health is not a safety problem, only a cost (Johansson 2009). Consequently, VZ does not call for a state of zero collisions, only zero deaths (Belin et al. 2012). By focusing on preventing crashes that cause fatalities and serious injuries, VZ creates a design mentality that emphasizes safety while allowing for some errors (Tingvall and Haworth 1999).

### Design principles of vision zero

The dominant safety strategy in road design has been to increase (and, when possible, to straighten) the physical space for drivers and cars, through the use of wider lanes and wider, straighter roads. The logic behind this is that if a driver runs off the road, a wider or straighter road allows for the driver to have more room to maneuver the vehicle back into the lane. Under VZ, these moves are viewed as undesirable because more space in the road contributes to higher speeds and, therefore, a driving environment in which injuries or fatalities are more likely (Johansson 2009).

This view of the trade off between collisions (which might be more likely in some circumstances when roads are narrow or have curves) and severe injuries is central to VZ. Under VZ, the ideal road system is one in which the human tolerance for mechanical forces is not exceeded – a concept originally proposed by William Haddon Jr. (Haddon 1970; Haddon 1980). According to Claes Tingvall, former Director of Traffic Safety in the Swedish Road Administration, the risk of injury from a road traffic crash can be viewed as an exponential dose-response relationship. If the mechanical forces (kinetic energy) that people face during road traffic crashes can be kept below the threshold for severe injuries, the road transport system can be considered safe (Tingvall and Haworth 1999).

Such thresholds are determined by assuming a certain level of vehicle safety. For example, well-designed cars are assumed to tolerate a maximum speed of 70 km/h for frontal impacts, and 50 km/h for side impacts (Tingvall and Haworth 1999). Studies have also shown that the survival rate is high when pedestrians are hit below 30 km/h (Anderson et al. 1997). Thus, these thresholds are used as a starting point for designing safer road systems under VZ.

The two main ways VZ tries to manage kinetic energy are by integrating compatible traffic components and by physically separating incompatible ones. Some examples include:

1. Vulnerable road users, such as pedestrians or cyclists, should not be exposed to vehicles at speeds over 30 km/h (18.6 mph). If separation is not possible, then reduce the vehicle speed to 30 km/h. Cyclists can reach these speeds, particularly on descents, and should also be separated from pedestrians or slowed.
2. Car occupants should not be exposed to other vehicles at speeds over 50 km/h (31.07 mph) in 90° crossings. If this is not possible, separate, reduce the angle (thereby altering the vector of force of the collision such that it reduces severe injury or death), or reduce the speed to 50 km/h.
3. Car occupants should not be exposed to oncoming traffic at speeds over 70 km/h (43.5 mph) if vehicles are about the same weight. If vehicles are of different weight, speeds should not exceed 50 km/h. If this is not possible, then separate traffic, balance automobile weights, or reduce speeds according to the maximum differential in vehicle weight.
4. Car occupants should not be exposed to the side of the road at speeds over 70 km/h, or 50 km/h if there are trees or other potentially dangerous objects. If this is not possible, separate cars from the side of the road or reduce speeds to 70 km/h or 50 km/h (according to road side conditions) (Johansson 2009).

“Separations” in this case are physical separations, such as crash barriers, tunnels, bridges, crossings at different levels, and different roads for different traffic vehicles, such as bicycle lanes. Temporal separation (e.g., traffic lights) is not considered a proper method of separation, and a space of just a few meters is not considered a spatial separation (Johansson 2009), such as when lines on the road are all that separate cyclists from traffic.

### Vision zero toolkit

VZ does not have a step-by-step manual on how to apply its philosophy and design principles; rather, it gives suggestions to system designers and safety planners on different methods they could utilize to attain a safer road system. While there is no one right way to implement VZ, some common road design elements have emerged in different VZ programs. This “toolkit” includes guidelines on how to optimize factors such as education, enforcement, and structural improvements, such as installation of median barriers, roundabouts, speed humps, and pedestrian islands. For illustration purposes, we briefly discuss a few of the structural changes recommended in the toolkit to illustrate the benefits and challenges of implementing core design features of VZ.

#### Median barriers

Cross median crashes, which result when a vehicle crosses into oncoming traffic, are one of the most severe types of crashes because of the high speeds involved and the risk of head-on collisions (Chitturi et al. 2011). One way to prevent such crashes is by installing median barriers. There are three main types of median barriers: rigid barriers, semi-rigid barriers, and cable barriers.

A common type of rigid median barriers are concrete barriers. Even though the installation cost of concrete barriers is more expensive than other types of median barriers, they are often used due to their relatively low life-cycle cost and maintenance-free characteristics (U.S. Department of Transportation Federal Highway Administration 2015). They have also proven to be extremely effective in preventing cars from crossing the median into oncoming traffic, especially in areas with high traffic volumes and high speeds. However, crashes involving concrete median barriers are associated with severe injuries. This is because rigid barrier systems absorb the least amount of kinetic energy in crashes, transferring the energy to road users in traffic collisions (U.S. Department of Transportation Federal Highway Administration 2015).

In contrast, both cable and semi-rigid barriers (e.g., guardrails) are much more forgiving since these barriers absorb most of the energy during collisions (U.S. Department of Transportation Federal Highway Administration 2015; Alluri et al. 2014; Hu and Donnell 2010). In fact, one study found that the odds of injury, as compared to hitting a hazardous object (e.g., utility pole, tree, wall, building, etc.), were reduced by 39% when hitting a concrete median barrier, 65% when hitting a guardrail, and between 78 and 85% when hitting a cable median barrier. The study also found that the odds of injury when hitting a guardrail were 43% lower than when hitting a concrete median barrier, and the odds of injury when hitting a near-side median cable barrier were 57% lower than when hitting a guardrail (Zou et al. 2014). These results suggest that cable median barriers are effective in minimizing harm.

However, while installation of cable median barriers decreases crash severity, it actually increases collision frequency, highlighting the importance of systems thinking within Vision Zero. For example, a meta-analysis of the safety value of median barriers concluded that cable median barriers increase the crash rate by 30%, but reduce the chance of sustaining a fatal and personal injury when in a crash by 20 and 10%, respectively (Elvik 1995). In Washington state, the total number of crashes in medians almost doubled when cable median barriers were installed (Ray et al. 2009). The installation of cable median barriers was also found to increase single-vehicle crashes on wide, depressed medians by 70% (Villwock et al. 2011). Since cable median barriers tend to increase crash frequency, they should not be considered as a default option.

Specifically, concrete barriers might be superior to cable median barriers in environments where barriers are frequently hit by vehicles, since guardrails and cable barriers require more maintenance after a crash (U.S. Department of Transportation Federal Highway Administration 2015; Zou et al. 2014). Additionally, the type of vehicle common to the area is important to consider since odds of injury among motorcyclists have been found to be greater in crashes with w-beam guardrails than with concrete median barriers (Daniello and Gabler 2011). This means that concrete median barriers may be preferred in low-income settings where motorcycles or motorbikes are the dominant mode of transportation. Furthermore, if autonomous vehicles become more common, installation of cable median barriers may present challenges since autonomous vehicles may have greater difficulty detecting smaller objects, such as cable median barriers. There is a strong need for researchers to develop mathematical models that provide policy feedback given characteristics such as crash history, ability to repair and maintain barriers, road user mix, and compatibility with existing infrastructure.

#### Modern roundabouts

Many injury crashes occur in intersections because of the high concentration of vehicles and pedestrians at these locations. There are numerous ways to try and control these intersections (e.g., all-way stops, two-way stops, traffic signals, etc.) (Ewing and Dumbaugh 2009). Depending on the circumstances, some methods are more effective than others at reducing the incidence and severity of traffic injuries. One method that has become favored under the VZ model is the use of modern roundabouts because they tend to decrease the kinetic energy transfer in collisions (Swedish Transport Administration 2012). They accomplish this by both slowing down vehicle speeds and removing specific types of collisions, such as right angle crashes and left turn head-on collisions (Ewing and Dumbaugh 2009). Specifically, the three basic principles of modern roundabouts – yield at entry, traffic deflection, and curvature of the travel path – all work in conjunction to reduce travel speeds, while the counter-clockwise circulation of vehicles eliminates many conflict points (Ewing and Dumbaugh 2009).

A meta-analysis study of non-US reports also showed that modern roundabouts are associated with a 30–50% reduction in the number of injury crashes, and a 50–70% decrease in the number of fatal crashes (Elvik 1847). In the US, using data from before-and-after the installation of 24 modern roundabouts, there was a 76% reduction in injuries and an 89% reduction in fatalities (Retting et al. 2001). A Swedish study found that the observed number of pedestrian crashes on single-lane roundabouts was 3–4 times lower than what was predicted, which suggests that single-lane roundabouts should be preferred over other designs in pedestrian-heavy areas (Brude and Larsson 2000).

On the other hand, when subgroup analyses are conducted, modern roundabouts seem to be effective only among certain populations. This may be because modern roundabouts add a dimension of complexity that, while slowing the driver, may also frustrate and distract the driver. For example, one study found that modern roundabouts increase the number of injury crashes involving bicyclists by 27% (Daniels et al. 2008). Another study found that replacing traffic lights with modern roundabouts increases the number of injury crashes involving vulnerable road users (i.e., pedestrians, cyclists, moped drivers, and motorcyclists) by 28%. This is especially alarming because vulnerable road users are already more likely to be fatally or seriously injured in crashes (De Brabander and Vereeck 2006). Therefore, depending on user and road characteristics, installation of modern roundabouts may be more harmful than beneficial.

Nevertheless, while there are some specific findings that suggest otherwise, most studies on modern roundabouts seem to point to a positive effect. This is especially true in European countries where roundabouts are very common (Ewing and Dumbaugh 2009). Other countries that are not as accustomed to modern roundabouts, however, may confront unintended consequences from installing these circular intersections. For example, the United States has only had limited experience with using modern roundabouts (Retting et al. 2001). In turn, many people mistake them for traditional traffic circles (Ewing and Dumbaugh 2009). This is potentially dangerous because drivers typically enter traffic circles at around 30 mph, whereas modern roundabouts are designed for speeds of up to only about 15 mph (Retting et al. 2001). If people in the region do not know how to properly use modern roundabouts, then the road system may become more dangerous after their installation. It is important to take note of the differences in traffic culture before installing modern roundabouts at intersections.

#### Speed humps

Speed humps are raised sections of pavement that are usually built from curb to curb. They are approximately 12 ft long and have a maximum height of 4 in. (Ben-Joseph 1995). Speed humps help reduce vehicle speeds by forcing drivers to slow down. If they don’t, the hump exerts a vertical force on the vehicle—the faster the vehicle is moving, the stronger the force. The discomfort felt by the drivers helps encourage them to lower their driving speed. Speed humps are normally applied in residential neighborhoods; along with decreasing speed, they also help increase the safety of residential streets, improve the quality of residential neighborhoods, and improve the traffic flow in residential areas (Fazzalaro 2006).

Speed humps have been found to be efficacious in terms of harm minimization, and are especially appropriate in areas with a high concentration of vulnerable road users. One study found that within residential neighborhoods, living within a block of a speed hump was correlated with almost a 2-fold reduction in the odds of injury. This protective effect was even greater for children; children living within one block of a speed hump showed a 2.5-fold reduction in the odds of injury (Tester et al. 2004). Another study found that the average reduction in injury collisions attributable to speed humps (44%) was twice that of sites where only speed enforcement cameras were used to control speeds. Furthermore, speed humps were the only type of element found to significantly decrease the number of fatal and serious crashes as compared to speed enforcement cameras and various narrowing or horizontal deflections (Mountain et al. 2005).

However, the installation of speed humps is not ideal for all situations, such as on bus and truck routes (http://www.nyc.gov/html/dot/html/pedestrians/traffic-calming.shtml; Parkhill et al. 2007). Variations to the standard speed hump design have also been created to better accommodate local infrastructure. For example, New York City implemented speed tables, which are speed humps with flat tops, as a traffic calming measure. However, speed tables were found to not reduce the total number of crashes (Ewing et al. 2013). Some system designers have also shown concern over the fact that speed humps may increase the amount of time it takes for an emergency response vehicle to reach its destination. In response, speed slots and speed cushions were created. Speed slots and speed cushions are similar to speed humps in that they are raised platforms that stretch across roads with the aim to reduce vehicle speeds. However, speed slots and speed cushions have separations in the humps so that emergency vehicles can avoid the humps when necessary (Johnson and Nedzesky 2004). More research needs to be done to determine the safety efficacy of these methods and the road and user characteristics for whom speed humps are safest.

#### Pedestrian islands

About 12% of all traffic fatalities annually in the US are from pedestrian crashes. Over 75% of these fatalities occur in non-intersection areas (U.S. Department of Transportation Federal Highway Administration 2013). Many of these deaths, however, are preventable by installing raised medians and pedestrian islands at these non-intersection areas. Pedestrian islands allow pedestrians to cross in two stages: once at the curb and again at the center island. Pedestrian islands, if properly used, provide a refuge so that pedestrians have a safe place to wait before crossing the second half of the street. This is especially helpful for pedestrians who walk at slower speeds (e.g., elderly people) since they now have the time to cross one direction of traffic at a time (Retting et al. 2003a). Additionally, it has been shown that sites without central refuges experience traffic delays because of the close proximity of vehicles to pedestrians (Griffiths et al. 1984). Thus, pedestrian islands are a method to separate pedestrians and vehicles by space so that various road users do not come into physical contact with one another. This means that pedestrian islands and raised medians provide a safe haven for people, while simultaneously increasing the traffic flow of the road system (Bowman and Vecellio 1994a).

One study found that refuge islands lower the risk of pedestrian crashes by up to two-thirds (Garder 1989). Another study found that pedestrian crash rates are much lower on multilane roads with raised medians than on those without raised medians (Zegeer et al. 2005). A different study on suburban and urban median types found that arterials with raised medians had the lowest pedestrian crash rate in suburban areas. Additionally, the pedestrian crash rate for arterials with raised medians were lower than both two-way left turn median lanes and undivided cross sections in central business district areas (Bowman and Vecellio 1994b).

A significant number of pedestrians, however, fail to actually wait on a refuge island, and instead dash across the road. For example, one study found that only 23% of pedestrians actually waited on the island. The remaining 77% who chose to cross the street without stopping on the island increased the risk of injury from a pedestrian-motor vehicle collision by not crossing with the pedestrian signal. Additionally, inclement weather caused compliance rates to drop even further; only 10% of pedestrians complied with the two-stage crossing in cold weather, as compared to 23% in warm weather (Li and Fernie 2010). While safety may be compromised due to non-compliers at pedestrian islands with crosswalk signals, more information is needed on which socio-demographic characteristics predict compliance and whether they work best in combination with other measures.

The “noncompliance” of pedestrians at refuge island crossings highlights the importance of road user compliance more generally. The use of road safety features is probably determined by an array of local characteristics (demography, weather, existing road design) that require more research.

### Enforcement under vision zero

As noted throughout this paper, VZ shifts the emphasis from the “unsafe driver” to the “unsafe road” (Elvebakk 2007). However, since VZ takes into account the entire road system, only focusing on the design and construction of safer roads is not enough. Traffic crashes have a multitude of causes; different enforcement strategies need to be implemented to complement road design elements. Some examples of enforcement techniques proposed under VZ include technological improvements in motor vehicles, installation of speed cameras, the use of red light cameras, and the professionalization of road users (Elvebakk 2007; City of New York 2014; Walljasper 2015; http://www.visionzeroinitiative.com/; Swedish Transport Administration 2012). There have been great innovations in enforcement since VZ was first discussed in the literature, and we briefly explore them here.

There are a variety of technological innovations that have the ability to enhance the safety of vehicles. One suggested idea is the integration of seat-belt ignition interlocks, which prevent the car from starting unless the occupants are buckled up. More controversial technologies include alcohol interlocks, which prevent the car from starting if the driver is over the legal alcohol limit, Intelligent Speed Adaptation systems, which support drivers in complying with the speed limit through alerts or by automatically correcting the vehicle’s speed, and black boxes, which are event data recorders (Elvebakk 2007). While these enforcement strategies are deemed paternalistic by some, advocates of these measures argue that they are justified due to the negative externalities associated with traffic crashes (Latour 1993). In other words, people who break traffic laws often expose other road users or pedestrians to significant injury risk, as well as burden society with substantial financial costs (Elvebakk 2007). The development of new technologies may help to ensure that road users are behaving appropriately when behind the wheel.

Mobile and fixed speed cameras also encourage the adoption of VZ principles through speed control. One study conducted in the UK demonstrated that the use of mobile speed cameras caused a 51% decline in injury crashes at distances of up to 500 m from the camera site (Christie et al. 2003). A second study in the UK found that fixed speed cameras were effective at lowering mean speeds on 30 mph roads by 4.4 mph and the percentage of drivers exceeding the speed limit by 35%. This led to a 20% average reduction in collisions 1 km upstream and downstream from the camera (Mountain et al. 2004). Additionally, a time series analyses performed in Barcelona showed that fixed speed cameras were effective in reducing the number of crashes and people injured in medium to high-speed roads, though the effectiveness cannot be generalized to roads with lower speed limits and traffic lights (Novoa et al. 2010). Therefore, system designers may want to consider installing speed cameras in strategic locations in order to nudge road users to follow the speed limits.

Data show that motorists are more likely to be injured in crashes that involve running a red light than in any other type of urban crashes. In response to this, red light cameras are increasingly being used to help communities enforce traffic laws by automatically photographing vehicles that run red lights (Retting et al. 2003b). In an analysis of motor vehicle crash data from Oxnard, California—one of the first US communities to employ red light cameras—Retting and Kyrychenko (Retting and Kyrychenki 2002) found that the installation of red light cameras reduced the number of crashes at signalized intersections and injury crashes by 7 and 29%, respectively. Additionally, the study found that right-angle crashes, which are associated with red light violations, were diminished by 32%, and right-angle crashes involving injuries were diminished by 68%. Of note, red light camera enforcement has led to an overall reduction in injury crashes by 25–30%, but has led to an increase in the number of rear-end crashes (Retting et al. 2003b). However, red light cameras still adhere to VZ principles since VZ focuses on decreasing health loss rather than on the number of crashes.

Taking a systems approach is just as central to enforcement elements of VZ principles as structural elements. But a systems approach is also difficult to accomplish, due to the large number of independent actors who enter and leave the road system.

Unlike the road transport system, many of the actors in the air or railway industries are professional operators. They act a certain way in their designated systems at least, in part, because they are paid to do so; they are paid to follow the instructions laid out by their employers. The majority of road users, on the other hand, act as individuals. Consequently, this makes it extremely complicated to regulate behaviors, especially since interventions in the private sector are seen as very intrusive by drivers (Elvebakk 2007).

Nonetheless, successful implementation of VZ probably requires an increase in monitoring road users, which the Swedish Road Authorities (SRA) have started doing by experimenting with alcohol and seat belt interlocks for professional drivers. The SRA claim that 40% of transport work undertaken on Swedish roads is from professional drivers, thus professional drivers should be placed at the forefront of road safety. Additionally, employers should introduce restrictions to protect their employees, such as regulating the use of electronics (i.e., mobile phones, radios, etc.) while driving and mandating rest periods. Ultimately, the professionalization of road users helps to introduce larger safety margins into the road system so that the likelihood of suffering an injury from a crash is further diminished (Elvebakk 2007).

It is important to note that traffic enforcement will need to be continually developed as the road transport system advances. For example, an increase in the uptake of autonomous vehicles in the near future will have significant effects on traffic policing, both positive and negative. One advantage to autonomous vehicles is that they are a potential method to regulate the road system by programming cars to adhere to the speed limit. Additionally, autonomous vehicles can ensure that no automobile runs a red light. However, autonomous vehicles can also disrupt policing, especially when there is an amalgam of human drivers and autonomous vehicles on the road as it could become difficult for police to detect which cars are being operated by people. Therefore, autonomous vehicles present both advantages and disadvantages when it comes to enforcing traffic regulations.

## Conclusion

VZ introduces a new paradigm for road safety: safety cannot be traded for mobility. Road users should not have to risk facing death every time they enter the road transport system. However, VZ also does not solely promote the lowering of speed limits. Instead, VZ demonstrates how mobility and safety can be jointly promoted if design principles are properly applied. This is evidenced by the VZ toolkit, which provides examples of existing traffic design elements that can improve traffic flow while simultaneously decreasing the injury crash rates. By applying the appropriate tools, road users can arrive at their destinations in a timely manner without getting hurt.

VZ also emphasizes the importance of shared responsibility. No longer is the individual fully responsible for road safety, but system designers should also be held accountable. This radical new perspective allows for the construction of a more integrated road system that is built around tolerating human error. It is important to note, though, that individual road users are still partly responsible for safety under VZ. While system designers have the ability to solve design and infrastructure-related problems, road users must adhere to the regulations set by system designers in order for the road transport system to actually become safer. Public health scientists need to collaborate with urban planners and city officials to build better data systems that can predict the optimal mix of road and enforcement characteristics given the mix of users.

In all cases, autonomous vehicles represent a wildcard in the VZ scenario that must be taken into account when deciding which measures to implement and which to ignore. Certainly, more extensive models are needed to account for the presence of such vehicles on the road as localities increasingly adopt VZ. But these models need to be integrated with the onboard predictive analytics offered by private corporate entities if they are to be optimized. Never before has buy-in from industry been more important.

Ultimately, VZ is a balancing act regarding road safety – the balance between safety and mobility, system designers and road users, traffic design and enforcement, and traditional engineering and public health. Under VZ, road safety becomes a collective, public health problem that needs to be addressed starting with how society views the road transport system. All in all, VZ changes people’s perceptions of the road transport system from one that is inherently dangerous to one that is safe and only risky if the system is not functioning properly (Elvebakk 2007).

## Abbreviations

SRA: Swedish road authorities
VZ: Vision zero
